# Beyond the Handicap: A Cross-Sectional Study of Mental Health Amongst Registered Golfers in Spain

**DOI:** 10.3390/sports13030080

**Published:** 2025-03-07

**Authors:** P. Martín-Escudero, M. Fuentes-Ferrer, F. Peinado Miguel, E. Jiménez-Herranz

**Affiliations:** 1 Department of Radiology, Rehabilitation and Physiotherapy, Medical School of Sport Medicine, Faculty of Medicine, Complutense University of Madrid, 28040 Madrid, Spain; mariaelj@ucm.es; 2 Research Unit University Hospital Nuestra Señora de Candelaria, Canary Health Service, 38010 Santa Cruz de Tenerife, Spain; mfuentesferrer@gmail.com; 3 Preventive Medicine Department, University Hospital Nuestra Señora de Candelaria, Canary Health Service, 38010 Santa Cruz de Tenerife, Spain; 4 Department of Journalism and Global Communication, Faculty of Information Sciences, Complutense University of Madrid, 28040 Madrid, Spain; peinado@ucm.es

**Keywords:** golf, physical activity, mental health, sports habits, GHQ-28 (General Health Questionnaire), cross-sectional study, Spain, sociodemographic factors, prevalence, sports players

## Abstract

The prevalence of impaired mental health (MH) has increased in recent decades. The aim of the study was to analyze the prevalence of impaired MH amongst registered Spanish golfers using the GHQ-28 questionnaire. A cross-sectional online survey was carried out in Spain between March and August 2024 using a self-administered questionnaire on 298 adult participants. The participants were mainly men (75.1%) over 60 years of age (50.7%) who were married or with a partner (79.5%), and who were active in the job market (56%). Of the participants, 73.5% reported an improvement in MH levels attributed to the game, and 77.2% recommended golf as a way to improve MH. The prevalence of impaired MH was 6.7% (95% confidence interval [CI]: 4.1–10.2%). Differences in impaired MH were found according to age (<=45 years: 28.6%; 46–60 years: 5.0% and >60 years: 4.0%; *p* = 0.001), marital status (married or with a partner: 4.6% vs. other: 14.8%; *p* = 0.009) and time of play (morning: 3.3% vs. other: 10.3%; *p* = 0.015). The prevalence of impaired MH detected was low and these results indicated that such playing habits and characteristics may have a positive impact on the mental wellbeing of golf players.

## 1. Introduction

Mental health is understood as a state of wellbeing that enables people to cope with stress, develop skills, and contribute to their community. However, its scope is not limited to the absence of mental illness, and it lacks any universally accepted definition [1,2]. The WHO describes it as essential for general health and for the capacity to make decisions, relating to others and taking action in social settings [3]. Mental health can be conceptualized as a field defined by intersecting axes corresponding to mental wellbeing and mental illness. Research has identified a correlation between low reported wellbeing and symptoms of anxiety and depression. Consequently, it is important to identify individuals experiencing mental illness so that they can enjoy a state of mental wellbeing and thus lead a meaningful life and contribute to society [4]. The most common disorders, such as depression and anxiety, affect millions of people worldwide and have increased in the last 25 years, representing a significant cause of disability [5,6]. Recent studies in Spain indicate that 10% of the adult population present mental health issues. Women tend to be more affected by this problem than men, and 10.7% take medication for mental disorders, especially anxiolytics and sedatives [7].

A person’s mental health depends on biological, psychological, and environmental factors, and requires an integrated approach to offer effective results [2]. Regular physical exercise has been identified as an effective strategy, not only for physical health but also to reduce the risk of depression and anxiety, improve sleep patterns, mood, self-esteem and overall psychological wellbeing [8,9]. Furthermore, sports develop resilience, strengthen social relationships and improve cognitive functions at all stages of life, helping to improve memory, learning and self-perception [10,11]. The relationship between sport participation and its influence on mental health is associated with the existence of neurochemical changes. An increase in the production of neurotransmitters (serotonin, noradrenaline, glutamate and endorphins) that modulate mood and stress has been described. Furthermore, neurophysiological changes in the gut–muscle–brain axis network have shown evidence of improved neuronal survival, resilience to physical aggression in the brain, cognitive function, cerebral vascularization, neuroinflammatory stability, resistance to the effects of ageing, neuroplasticity, neurogenesis and synaptic function in brain regions such as the hippocampus [12,13].

Golf is an open-air sport in which players strike a ball with specific clubs to complete holes in the lowest possible number of strikes. It has alternative modalities such as par 3 golf and indoor simulators [14]. Although this activity involves moderate physical activity, the requirements of the game can vary according to factors such as walking on the course, the topography and the player’s own characteristics [15]. Golf is growing in popularity around the world. Golf participation may contribute to increased life expectancy and reduced mortality. This sport enables individuals to meet and exceed minimum health recommendations for physical activity, contributing to improving aerobic fitness and reducing cardiovascular risk factors. Furthermore, improvements in other physical capacities such as proprioception, balance, strength, and muscle function have been reported, particularly in older adults. Benefits have also been observed in respiratory system and metabolic function. This sport has an overall positive impact on wellness and mental ill health, favoring the creation of a social meeting place, improved focus and concentration, practical help and support and reduced mental symptom burden. Golf also provides opportunities for intergenerational interaction and social connections. The physical and cognitive demands inherent in this sport are the basis for these potential improvements in health status [16,17,18,19,20]. The influence of psychological resources, such as self-compassion and wellbeing, on golfers’ sport performance has also been investigated, although no short-term associations have been identified [21]. The promotion of golf as physical exercise has also been recommended in public health policies to improve the population’s mental and physical health [20,22].

The main aim of this study is to determine the prevalence of impaired mental health amongst registered golf players. Secondary aims include establishing sociodemographic factors and golf playing characteristics associated with the prevalence of mental health, and to describe the players’ own perceived mental health benefits from golf to reflect the interest of this sport as a good ally for better mental health. Previous studies have described golfers exhibit lower levels of cognitive and somatic anxiety compared to non-golfers [23]. Furthermore, golf participation has been positively associated with improved physical activity levels, social trust and personal wellbeing [20,24,25].

## 2. Materials and Methods

This is a descriptive, cross-sectional observational study with information gathered from March to August 2024. Registered golfers from all autonomous communities in Spain, of all genders, in age groups from 18 to 75 years were included in the study and had to give consent to participate in the study. The study was approved by the Committee for Ethics in Research with Medical Products (CEIm, by its Spanish acronym) of the Hospital Clínico San Carlos Madrid, Spain.

Participants were recruited via non-probabilistic convenience sampling, using a range of media, social networks and snowball sampling. Information was given on the aims of the study and instructions on how to participate in it were provided via a link to the study.

An online epidemiological questionnaire was designed on the EU Survey platform for gathering the data accessed on 20 April 2024 (https://ec.europa.eu/eusurvey/runner/7dcfb602-b35c-e50c-6fad-be13ead80041). It included sociodemographic variables (age, sex, autonomous community of residence, qualifications, marital status and current job situation), golf playing habits (place where participant plays, competitive level, playing experience, playing time and frequency, modality and type of course) and other sporting activities (type and frequency of sport per week). Variables on the perceived effect of golf on mental health were included, along with recommendations to play it in order to improve health. These were evaluated using a 5-point Likert scale. The questionnaire included the Spanish validated version of Goldberg’s General Health Questionnaire (GHQ-28). The questionnaire contained a total of 28 questions divided into 4 sub-scales that evaluated four dimensions, with 7 questions for each one (A: questions 1 to 7, B: questions 8 to 14, C: questions 15 to 21, D: questions 22 to 28), referring to somatic symptoms, anxiety/insomnia, social dysfunctions and major depression, respectively. Each question had four possible single answers. The score chosen for this study was the one that assigned the values 0, 0, 1 and 1 to the answers of the items in the order established by the questionnaire. The main variable of the study was the total score from the GHQ-28, in which impaired mental health was considered with scores of >=6 [26].

The number of registered golfers as of 31 December 2023 (data provided by the Royal Spanish Golf Federation) was 297,378. For an approximate participation of 300 participants, a confidence level of 95% and an expected standard deviation in the total score of the GHQ-28 questionnaire of 9.01 [27], a precision of 1.02% would be obtained. The calculation was performed using the statistical program EPIDAT version 3.1.

The qualitative variables were presented with their absolute and relative frequencies. The prevalence of impaired mental health was estimated (GHQ-28 >= 6) along with the confidence interval at 95%. The association of the qualitative variables with the prevalence of the scores in the GHQ-28 >= 6 was analyzed with the chi-squared test or Fisher’s exact test, along with the odds ratio (OR) and its confidence interval (CI) at 95%. The null hypothesis was rejected for a bilateral significance value of 5% for all hypotheses testing. Data obtained were analyzed using SPSS Statistics version 26 (IBM Corp. Released 2019. IBM SPSS Statistics for Windows, Version 26.0. IBM Corp., Armonk, NY, USA).

## 3. Results

The total number of participants in the study was 298. They all voluntarily agreed to participate in the study, giving their informed consent before the survey was taken. All the subjects were over 18 years of age, the majority of which were residents in the Autonomous Community of Madrid (Spain).

Table 1 shows the subjects’ sociodemographic and descriptive data, their golf practices, and the results of the relation between sociodemographic variables, playing golf and other sports and the prevalence of impaired mental health (GHQ-28 >= 6). The sociodemographic variables showed a statistically significant drop in the level of impaired mental health among subjects who were married or had a partner (OR: 0.28; 95% CI: 0.11–0.72; *p* = 0.009) and among participants in age groups over >45 years. In particular, participants in the age groups 46–60 and >60 years had a lower probability of impaired mental health compared to the group <=45 years (OR: 0.13; 95% CI: 0.04–0.42; *p* < 0.001, OR: 0.10; 95% CI: 0.03–0.33; *p* < 0.001, respectively). As regards the variables for golf playing, a significantly lower prevalence of impaired mental health was found among golfers who played in the morning (OR: 0.29; 95% CI: 0.104–0.83; *p* = 0.015), while an insignificant prevalence was found among subjects who had played golf for >= 4 years (OR: 0.42; 95% CI: 0.14–1.22; *p* = 0.158). Playing other sports, and the type and frequency of sport, were not related to impaired mental health.

A total of 201 subjects (67.4%) stated that they played other sports. The highest frequency for playing other sports was <2 days/week (100 (49.8%)), followed by 2–4 days/week (82 (40.8%)) and >4 days/week (29 (9.5%)). The sports most frequently played were those classified as aerobic or endurance exercises (107 (35.9%)), followed by mixed sports (89 (29.9%)), while anaerobic sports were most infrequently played (5 (1.7%)).

**Figure 1:**
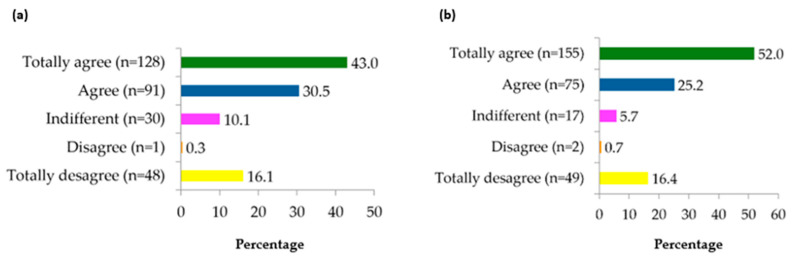
Description of the responses reported by participants regarding issues related to golf practice and mental health. The results of the different responses to the question about personal perceptions of the links between playing golf and its effect on mental health (**a**) and recommendations to play golf for its positive effect on mental health (**b**).

The study on the personal perceptions of playing golf and its relation to mental health showed that 73.5% (219) felt an improvement in their mental health thanks to golf. This group included subjects who answered affirmatively with “totally agree” and “agree”. As regards recommendations to use golf as a way to improve mental health, 77.2% (230) would recommend golf to improve this aspect of a person’s health. This percentage included subjects who answered affirmatively with “totally agree” and “agree”. These data can be seen in Figure 1.

**Figure 2:**
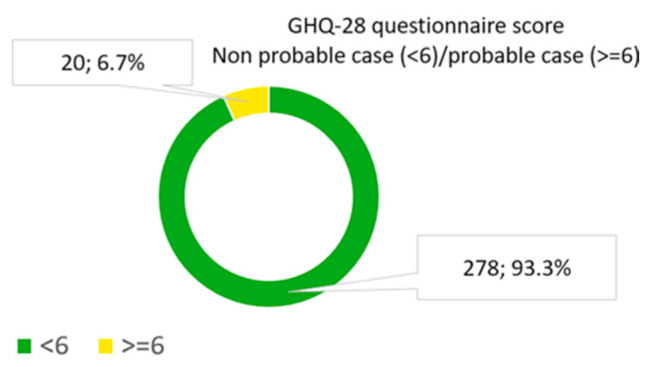
Data on mental health according to the score obtained in the GHQ-28 questionnaire.

The prevalence of impaired mental health (GHQ-28 >= 6) in our sample was 6.7% (CI 95%: 4.1–10.2) (Figure 2).

**Figure 3:**
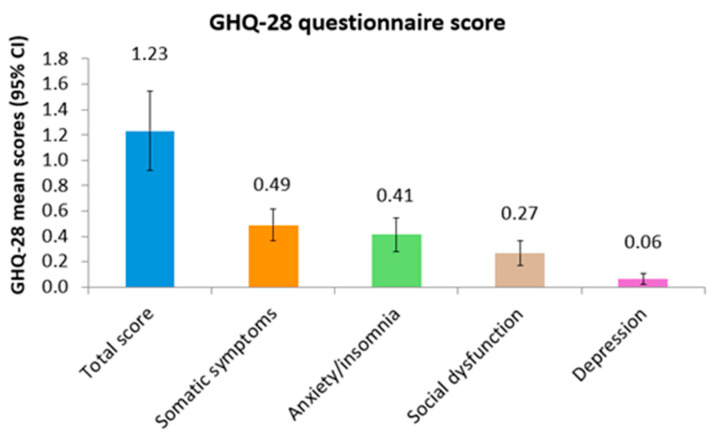
Results of the GHQ-28 questionnaire scores (mean and 95% confidence interval).

Figure 3 shows the total scores (mean and 95% confidence interval) for each of the four sections of the questionnaire. Symptoms of depression obtained the lowest scores, while the highest ones were in somatic symptoms.

A statistically significant number of positive recommendations for golf as a way to improve mental health was found among golfers who presented impaired mental health in comparison to those who presented no impairment (8.3% (totally agree/agree) vs. 1.5% (rest), *p* = 0.034). A similar result was found among subjects who felt that playing golf had improved their mental health (8.2% (totally agree/agree) vs. 2.5% (rest), *p* = 0.083).

## 4. Discussion

A 6.7% prevalence of impaired mental health was found in this study among registered golfers in Spain. The factors associated with a lower prevalence of impaired mental health were occupying the >45 year age group, being married or with a partner and playing golf in the morning.

Our sample showed a profile for a recreational golfer of a male over 45 years of age, married and active in the job market, a similar profile to the one found in other research [28,29]. Golf is regarded as suitable for persons of all age groups and physical conditions and is beneficial for health at any time of life [20]. Elite/sub-elite sports players, including golfers, have been described as presenting more mental health issues, such as anxiety and distress [23,30,31]. In our sample, we found a higher but not significant prevalence of impaired mental health amongst elite/sub-elite golfers, who represented only 5% of the sample in this study. More research into this area is required, using specific assessment instruments for elite athletes, such as the ones developed by the International Olympic Committee [31,32].

Golf is generally presented as an activity that has potential benefits for one’s physical, mental and social health in a range of populations [16,22,25,33]. This study showed a prevalence of 6.7% of impaired mental health among golfers, with no distinction between genders, but with a decrease as the age of the participants increases. These results are lower than the ones reported in national and international surveys on the Spanish general public, which show prevalences between 16% and 18%, with opposing trends for age [7,34], although it should be borne in mind that other studies have found an increasing trend in impaired mental health among younger generations [35,36]. The differences we observed can be attributed to methodological, socioeconomic and educational factors, or to playing golf. Our study also found a lower prevalence of impaired mental health among married subjects and those with partners. These results coincide with other studies on the benefits of marriage for mental health [37,38]. Another aspect to consider is the potential impact of the COVID-19 pandemic on the mental health of various demographic groups, including athletes. Researchers have observed an increase in mental health issues such as anxiety and depression, as well as sleep disturbances and other somatic symptoms, among athletes in various sports. These clinical manifestations have been attributed to both the confinement period and infection [39,40]. Furthermore, the pandemic’s impact on the deterioration of sleep quality and quantity, as well as psychological wellbeing, has been observed in younger people [41]. There is a need for more in-depth research on the factors that influence mental health in different populations to enable more preventive and promotional measures to be taken, including the promotion of physical activities such as golf.

Two questions were used to evaluate the subjective perceptions of golfers regarding the benefits of this sport for mental health. The results showed a high percentage of affirmative answers, which were even higher among subjects whose scores indicated impaired mental health. This situation may be explained by the health belief model of behavior change, which suggests that an individual’s state of health can have an effect on their understanding of the benefits of physical exercise [42,43,44]. These findings imply that participants with higher scores in the GHQ-28 scale may be more likely to perceive the physical, psychological and social benefits of playing golf.

Previous research has shown the positive effects of golf on mental health, including improved physical conditioning, social contact, attention, concentration and quality of life, even for golfers with previous pathologies [19,20,24]. Analysis of the factors that can lead to golf having an effect on mental health shows significant evidence of a lower prevalence of impaired mental health among golfers who play in the morning, which, as some researchers suggest, may be an outcome of the influence of circadian rhythms on physical exercise [45]. A lower prevalence of impaired mental health was also found among golfers who had played for more years, more regularly, in more weekly games, on 18-hole courses and in the company of other golfers. These variables match the current ones in the model for prescribing physical exercise and the model for ‘mental health through sport’ [46,47]. The results of this study highlight the importance of the possible influence on different regions of the brain of physical exercise in a natural setting, with physical exercise accumulated over time and the social aspects that form a part of golf. Other studies have also shown these factors as important in the links between physical exercise and its benefits for mental health [12,13,48,49,50,51,52]. Another implication is that playing frequently, the number of years played, and continuity are also important factors, although more research is required to establish ideal parameters to prescribe golf as a physical exercise with benefits for mental health.

When analyzing the influence of other types of sport alongside golf on the prevalence of impaired mental health, no statistically significant differences were found between the sports played and their frequency. More than half of the participants did other sports, exceeding the volume of physical exercise carried out by the Spanish general population [34]. However, more in-depth studies are needed to examine the dose–response relationship of physical exercise in improving mental health, since there are no defined parameters and simply establishing an approach of ‘the more, the better’—commonly used when prescribing exercise for physical health—cannot be applied in this case [53].

This study on the mental health of registered Spanish golfers has some significant limitations and strengths. The main limitations include selection bias caused by the sampling method, the cross-sectional nature of the study, which stops causal relations from being established, and the limitations inherent to self-administered electronic questionnaires, since the sampling method used may have underestimated the actual value of mental health as only those who presented lower levels of impaired mental health were the ones to answer. Recruitment of participants was completed once the estimated sample size was reached; however, the analysis was conducted without two participants due to the missing information for most of the survey variables in these cases. Approximately half of the study sample is composed mainly of participants over 60 years of age, which limits the generalizability of the results found. Furthermore, the sample analyzed was made up mainly of recreational sports players, which limits the generalization of the results with regard to elite/sub-elite groups. The low percentage of impaired mental health detected did not allow for a robust multivariate analysis to be carried out. To our knowledge, this is the first national study of mental health prevalence in registered golfers. The study has been designed with a sample size to estimate impaired mental health with good precision, using validated questionnaires and in a sample of mainly recreational federated golfers. The study also includes a panel of sociodemographic variables, golf playing habits, practice of other sports and variables of perception of the effect of golf on the mental health of the participants.

## 5. Conclusions

Golf players, predominantly men over 45 years of age, married or with a partner and active in the job market, show a low prevalence of impaired mental health according to the GHQ-28 questionnaire, with lower scores in the depression sub-section and higher ones in the somatic symptoms sub-section. Most of the participants are recreational golfers with more than 9 years’ experience, who play golf 2–3 days a week in the mornings with other players. The participants state that sport has generally improved their mental health, and they recommend golf for this purpose. A lower prevalence of impaired mental health was observed among older players and among those who are married or with a partner, those who play golf in the morning in moderation with other players and those who have played for many years. These data suggest that such characteristics and habits when playing golf may have a positive impact on golfers’ mental wellbeing.

## Figures and Tables

**Table 1:** Sociodemographic and descriptive data for golf in the sample, and relationship between sociodemographic characteristics, playing golf and other sports and the prevalence of impaired mental health (GHQ-28 >= 6).

Variable	Total (n = 298)	No Impaired Mental Health (n = 278)	Impaired Mental Health (n = 20)	*p* *	Odds Ratio (CI 95%)
**Sex**					
*Male*	223 (75.1)	208 (93.3%)	15 (6.7%)		1
*Female*	74 (24.9)	70 (94.6%)	4 (5.4%)	0.781	0.79 (0.26–2.47)
**Age (years)**					
*<=45*	28 (9.4)	20 (71.4%)	8 (28.6%)		1
*46–60*	119 (39.9)	113 (95.0%)	6 (5.0%)	<0.001 *	0.13 (0.04–0.42)
*>60*	151 (50.7)	145 (96.0%)	6 (4.0%)	<0.001 *	0.10 (0.03–0.33)
**Active in job market**					
*Yes*	167 (56.0)	156 (93.4%)	11 (6.6%)	0.923	0.96 (0.38–2.38)
*No*	131 (44.0)	122 (93.1%)	9 (6.9%)		1
**Marital status**					
*Married/with partner*	237 (79.5)	226 (95.4%)	11 (4.6%)	0.009 *	0.28 (0.11–0.72)
*Other*	61 (20.5)	52 (85.2%)	9 (14.8%)		1
**Competitive level**					
*Elite/Sub-elite*	16 (5.4)	14 (87.5%)	2 (12.5%)		1
*Recreational*	282 (94.6)	264 (93.6%)	18 (6.4%)	0.292	0.48 (0.10–2.26)
**Time playing experience**					
*<4 years*	39 (13.1)	34 (87.2%)	5 (12.8%)		1
*≥4 years*	259 (86.9)	244 (94.2%)	15 (5.8%)	0.158	0.42 (0.14–1.22)
**Frequency**					
*<4 days/week*	263 (88.3)	244 (92.8%)	19 (7.2%)		1
*≥4 days week*	35 (11.7)	34 (97.1%)	1 (2.9%)	0.487	0.38 (0.05–2.91)
**Playing time**					
*<4 h*	164 (45.0)	153 (93.3%)	11 (6.7%)	1.000	1.00 (0.40–2.49)
*≥4 h*	134 (45.0)	125 (93.3%)	9 (6.7%)		1
**Time played last month**					
*0–3 days*	95 (31.9)	89 (93.7%)	6 (6.3%)	0.349	0.61 (0.21–1.74)
*4–8 days*	100 (33.6)	90 (90.0%)	10 (10.0%)		1
*≥9 days*	103 (34.6)	99 (96.1%)	4 (3.9%)	0.525	0.36 (0.11–1.20)
**Swing practice/course play**					
*Course+swing practice*	200 (67.1)	186 (93.0%)	14 (7.0%)		1
*Only course/only swing*	98 (32.9)	92 (93.9%)	6 (6.1%)	0.776	0.87 (0.32–2.33)
**Time of Play**					
*Morning*	153 (51.3)	148 (96.7%)	5 (3.3%)	0.015 *	0.29 (0.104–0.83)
*Other*	145 (48.7)	130 (89.7%)	15 (10.3%)		1
**Accompanied**					
*Individual*	48 (16.1)	43 (89.6%)	5 (10.4%)		1
*Group*	250 (83.9)	235 (94.0%)	15 (6.0%)	0.339	0.55 (0.19–1.59)
**Modality**					
*18-hole course*	238 (79.9)	225 (94.5%)	13 (5.5%)	0.143	0.44 (0.17–1.15)
*Other*	60 (20.1)	53 (88.3%)	7 (11.7%)		1
**Other sports (weekly)**					
*Yes*	201 (67.4)	187 (93.0%)	14 (7.0%)		1
*No*	97 (32.6)	91 (93.8%)	6 (6.2%)	0.801	0.88 (0.33–2.37)
**Classification of other sports**					
*No other sport*	97 (32.6)	91 (93.8%)	6 (6.2%)	0.543	0.72 (0.25–2.10)
*Aerobic*	107 (35.9)	98 (91.6%)	9 (8.4%)	0.711	1
*Anaerobic*	5 (1.7)	5 (100.0%)	0 (0.0%)	-	NE
*Mixed*	89 (29.9)	84 (94.4%)	5 (5.6%)	0.450	0.65 (0.21–2.01)
**Frequency of other sports**				0.476 0.643	
*<2 days week*	100 (49.8)	94 (94.0%)	6 (6.0%)	0.612	0.54 (0.10–2.92)
*2–4 days/week*	82 (40.8)	76 (92.7%)	6 (7.3%)	0.643	0.67 (0.13–3.62)
*>4 days week*	19 (9.5)	17 (89.5%)	2 (10.5%)		1

* Value of *p* < 0.05: statistically significant; NE: No estimable; CI: confidence interval.

## Data Availability

The questionnaire data are available on the following EU platform; data accessed on 20 April 2024: https://ec.europa.eu/eusurvey/runner/7dcfb602-b35c-e50c-6fad-be13ead80041.
